# Relation between red blood cell distribution width and acute kidney injury in patients with sepsis

**DOI:** 10.31744/einstein_journal/2022AO6828

**Published:** 2022-05-02

**Authors:** Marina Larissa Vettorello Ramires, Manoela Fidelis Batista Leite, Daniel Zu Yow Lo, Leonardo Bonilla da Silveira, Leonardo José Rolim Ferraz, Andreia Pardini, Araci Massami Sakashita, Andrea Tiemi Kondo, Guilherme Benfatti Olivato, Marcelino de Souza Durão, Adelson Marçal Rodrigues, Daniela Mendes Chiloff, Danilo Candido de Almeida, Miguel Angelo Goes

**Affiliations:** 1 Faculdade Israelita de Ciências da Saúde Albert Einstein Hospital Israelita Albert Einstein São Paulo SP Brazil Faculdade Israelita de Ciências da Saúde Albert Einstein, Hospital Israelita Albert Einstein, São Paulo, SP, Brazil.; 2 Hospital Israelita Albert Einstein São Paulo SP Brazil Hospital Israelita Albert Einstein, São Paulo, SP, Brazil.; 3 Universidade Federal de São Paulo São Paulo SP Brazil Universidade Federal de São Paulo, São Paulo, SP, Brazil.

**Keywords:** Acute kidney injury, Erythrocyte indices, Sepsis, Renal replacement therapy

## Abstract

**Objective:**

The objective of the present study is to evaluate the association of red blood cell distribution width with acute kidney injury in sepsis.

**Methods:**

This is a retrospective study of 849 critically ill patients with sepsis in intensive care unit. Demographic data, renal function, inflammation, complete blood count, and acid-base parameters were compared between acute kidney injury and non-acute kidney injury groups. Therefore, a multivariate analysis was performed to observe independent predictive factors.

**Results:**

Comparatively, higher levels of C-reactive protein, lactate, red blood cell distribution width, and Simplified Acute Physiology Score 3 were found in the acute kidney injury group. The study showed a higher frequency of women, hemoglobin (Hgb) concentration, platelets, bicarbonate and PaO_2_/FiO_2_ ratio in the non-acute kidney injury group. In addition, there was an independent association of comorbidity-chronic kidney disease [OR 3.549, 95%CI: 1.627-7.743; p<0.001], urea [OR 1.047, 95%CI: 1.036-1.058; p<0.001] and RDW [OR 1.158, 95%CI: 1.045-1.283; p=0.005] with acute kidney injury in sepsis patients.

**Conclusion:**

As an elective risk factor, red blood cell distribution width was independently associated with sepsis-related acute kidney injury. Thus, red blood cell distribution width acts like a predictive factor for sepsis-induced acute kidney injury in intensive care unit admission.

## INTRODUCTION

Acute kidney injury (AKI) is a common and serious syndrome with a high number of cases worldwide. Sepsis is the most important cause of AKI in critically ill patients with high mortality in the intensive care unit (ICU).^(1-4)^ Sepsis causes hypoperfusion with lower oxygenation and bicarbonate concentration but with higher lactate levels, with consequent organic dysfunction in critically ill patients admitted to the ICU.^(2-6)^

In line with this, sepsis-associated AKI is complex and multifactorial. It includes endothelial and tubule changes, infiltration of inflammatory cells, and intrarenal hemodynamic changes with intraglomerular thrombosis.^(3-6)^ In addition, AKI in sepsis may also be distinguished from non-sepsis AKI by some specific features and poor prognosis.^(2)^

Considering the hematological context, red cell distribution width (RDW) measures the amount of red blood cell variation in volume and size. The RDW is defined as the standard deviation of erythrocyte size divided by the mean corpuscular volume.^(7)^ In some cases, changes in RDW indicates blood-associated abnormalities or organ/systems dysfunction and acts as a predictor of outcomes in critically ill patients.^(8-12)^

In addition, RDW is a readily available biomarker related to decreased hemoglobin levels by inflammatory cytokines and impairing iron mobilization.^(7-10)^ Further, RDW can integrate various pathophysiological mechanisms associated with sepsis.^(8,9,11-13)^ Recently, researchers have reported an independent association of RDW and the risk of adverse outcomes in patients with sepsis.^(10,11)^ However, no evidence of the RDW association with sepsis-induced AKI was found. Therefore, this study analyzed the relationship of RDW and sepsis-induced AKI.

## OBJECTIVE

To analyze whether red blood cell distribution width could act as a predictor of acute kidney injury in patients with sepsis.

## METHODS

Medical records of patients with sepsis who were admitted to any unit of the Intensive Care Center of *Hospital Israelita Albert Einstein* (HIAE), São Paulo, Brazil, from January 1 to December 31, 2017, were retrospectively studied. First, the study observed that the patients were screened by quick Sequential [Sepsis-related Organ] Failure Assessment (qSOFA) for early suspected sepsis in the emergency department or bedside in the ward. A qSOFA ≥2 was considered altered with the following variables: Glasgow coma score <15, respiratory rate ≥22 breaths/minute, systolic blood pressure ≤100mmHg.^(14)^ Afterward, the patient was referred to the ICU.

Inclusion criteria were individuals over 18 years of age with a diagnosis of sepsis admitted to an ICU at HIAE. Patients with hematologic, advanced oncological disease, liver failure, chronic infection by HIV, hepatitis B and C, chronic kidney disease (CKD) stages 4 or 5, and patients who died within the first 24 hours of admission to the ICU were excluded.

The variables analyzed were age, sex, Hgb concentration, blood count parameters such as mean corpuscular volume, RDW, platelet, white blood cell count, serum levels of urea, creatinine, sodium, potassium, total bilirubin, C-reactive protein (CRP), arterial blood gas parameters, arterial lactate, arterial oxygen pressure/inspired oxygen fraction (PaO_2_/FiO_2_) ratio, and Simplified Acute Physiology Score 3 (SAPS 3) prognostic index.^(15)^ Outcomes such as AKI, need for Renal Replacement Therapy (RRT), red cell transfusion, mechanical ventilation, vasopressor drugs, and mortality were computed. Norepinephrine was the primary vasopressor used when mean arterial pressure was maintained <65mmHg after volume resuscitation. Continuous venovenous hemodiafiltration was used in patients with hemodynamic instability, and intermittent hemodialysis was used in stable hemodynamic patients who required RRT during the ICU period.

Acute kidney injury was defined and stratified according to the Kidney Disease Improving Global Outcomes (KDIGO) criteria, if there was an increase of 0.3mg/dL in 48 hours, or a 1.5-fold increase in serum creatinine in seven days, or diuresis lower than <0.5mL/kg/hour during six hours from ICU admission.^(16)^ A follow-up up to 28 days after admission to the ICU was conducted to determine the outcomes. This study was approved by the local ethics committee on human research at HIAE, São Paulo, Brazil (CAAE: 01520218.9.0000.0071; protocol: 3.025.097, CAAE: 02628918.7.0000.0071; protocol: 3.102.869 and CAAE: 93612218.2.0000.0071; protocol: 4.246.520). The study was conducted under the ethical standards of the responsible committee on human experimentation (institutional and national). Informed consent was waived, and researchers analyzed only not identified data.

### Statistical analysis

Categorical variables are presented as frequencies and percentages. Numerical data were described as mean ± standard deviation. In a comparative analysis, the χ^2^ test for frequencies was used. The Kolmogorov-Smirnov test was performed to find the normal distribution of continuous data. Afterward, the comparisons between the groups were made by the Student’s *t*-test for data with normal distribution and Mann-Whitney for non-parametric data. The Pearson correlation between two continuous variables was also performed, in addition to the binary logistic regression with the backward stepwise method for admission data using AKI as a variable response. The variables in the multivariate model were included when reached p<0.09. Regression data were expressed as odds ratios (OR) and 95% confidence intervals (95%CI). Moreover, the association by regression of ICU admission markers with the need for RRT and mortality was also determined. For that analysis, the marker variables from ICU admission that were independently significant with AKI were used. The statistical level p<0.05 was considered significant. Statistical analyses were performed in SPSS version 22 (IBM, Armonk, New York, USA) and Excel^TM^ 16.0 (Microsoft, Redmond, Washington, USA).

## RESULTS

**Figure 1:**
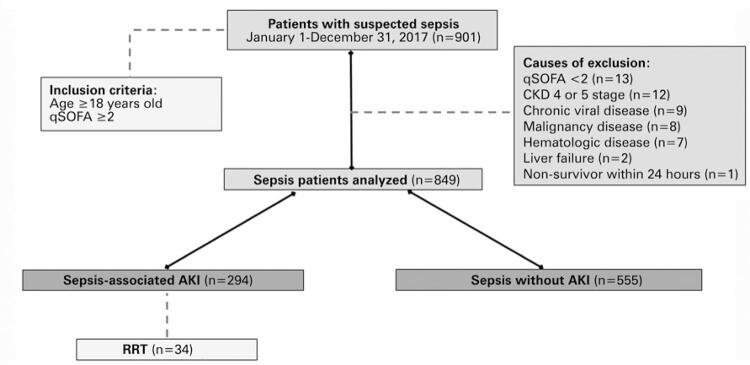
Workflow diagram of sepsis patient selection. A total of 901 patients with suspect of sepsis were in the intensive care unit at *Hospital Israelita Albert Einstein*, São Paulo, Brazil. After exclusion criteria the remaining patients (n=849) were subdivided in two groups: AKI Group and non-AKI Group. Afterward, renal replacement therapy with independent variables was analyzed variables

AKI: acute kidney injury; qSOFA: quick Sequential [Sepsis-related Organ] Failure Assessment; CKD: chronic kidney disease; RRT: renal replacement therapy.

**Figure 2:**
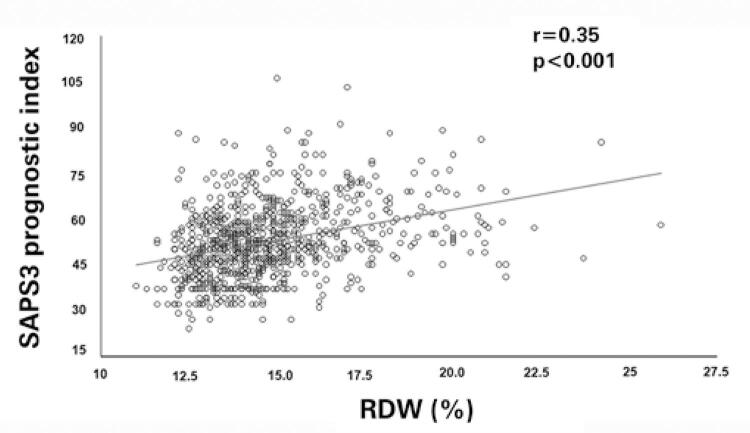
Correlation between simplified acute physiology score 3 and red blood cell distribution width in patients with sepsis

After exclusion criteria (Figure 1), the study was conducted with 849 patients with a diagnosis of sepsis. Classically, the patients were investigated about their primary site of infection. In these sepsis patients, 352 had respiratory tract infection, 183 had urinary tract infection, 139 had an undetermined primary focus, 105 had an abdominal infection, 50 had nervous system infection, and 20 patients had bloodstream infection as the focus of sepsis. Next, several admission variables of all patients were analyzed using multiple correlations. A positive correlation was found between age and SAPS 3 (r=0.57, p<0.001), SAPS 3 and RDW (r=0.35, p<0.001; Figure 2), RDW and urea (r=0.24, p<0.001), RDW and total bilirubin (r=0.24, p<0.001), RDW and age (r=0.21, p<0.001), RDW and lactate arterial (r=0.20, p<0.001), RDW and creatinine (r=0.18, p<0.001). Negative correlations were identified between bicarbonate and lactate concentration (r=-0.39, p<0.001), Hgb concentration and SAPS 3 (r=-0.34, p<0.001), Hgb and RDW (r=-0.32, p<0.001), bicarbonate and creatinine (r=-0.20, p<0.001), creatinine and Hgb (r=-0.16, p<0.001), and between Hgb and age (r=-0.15, p<0.001).

SAPS 3: simplified acute physiology score 3 prognostic index; RDW: red blood cell distribution width.

A positive correlation was found between simplified acute physiology score 3 and red blood cell distribution width in 849 patients with sepsis (p<0.001).

Furthermore, this study found that, among the total of sepsis patients, 294 had AKI diagnosis by KDIGO criteria. A total of 160 patients were in stage 1, 73 in stage 2, and 61 in stage 3, considering the KDIGO criteria. Thereafter, patients with sepsis were divided into two groups: AKI and without AKI (non-AKI) to perform several comparisons and associations.

**Figure 3:**
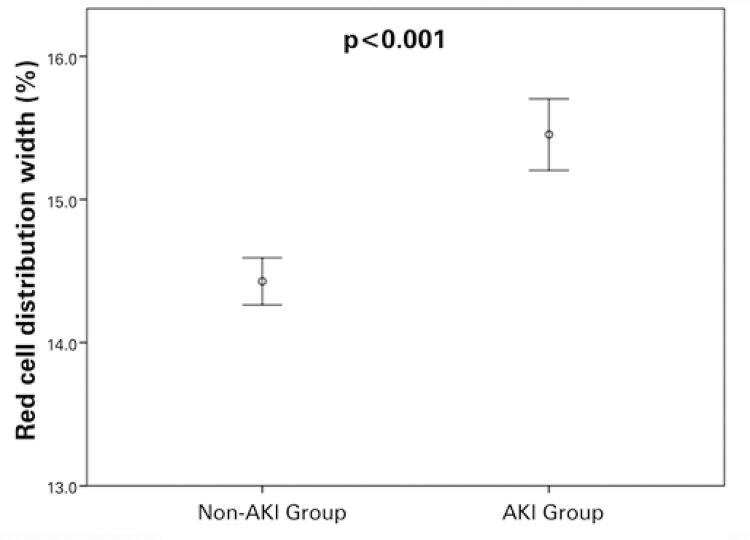
Comparison of red cell distribution width between acute kidney injury and non-acute kidney injury groups

Table 1 shows that the AKI Group had a higher frequency of men and comorbidities (*diabetes mellitus* (DM), hypertension, and CKD) compared to the non-AKI Group. Lower indices for mean arterial pressure, Hgb concentration, platelets, pH, pCO_2_, arterial bicarbonate, and PaO_2_/FiO_2_ ratio in AKI Group were found in comparison to non-AKI. Moreover, the AKI Group had higher mean age, and higher levels of creatinine, urea, total bilirubin, RDW (Figure 3), pO_2_, arterial lactate, and SAPS 3 prognostic index on admission to ICU compared to the non-AKI Group.

**Table 1:** Comparison of multiple variables on intensive care unit admission between acute kidney injury and non-acute kidney injury groups

Parameters	AKI Group (n=294) (%)	Non-AKI Group (n=555) (%)	p value
Sex			
Women	83 (28.2)	298 (53.7)	<0.001
Men	211 (71.8)	257 (46.3)	
Age (years)	76±15	69±19	<0.001
Comorbidity			
*Diabetes melittus*	109 (37.1)	131 (23.6)	<0.001
Hypertension	148 (50.3)	199 (35.9)	<0.001
CKD	75 (25.5)	15 (2.7)	<0.001
Smoker	59 (20.1)	100 (18)	0.47
MAP (mmHg)	78±21	86±19	<0.001
Creatinine (mg/dL)	1.9±0.7	1.1±0.4	<0.001
Urea (mg/dL)	82.6±42.9	44.6±17.8	<0.001
Sodium (mEq/L)	135±13	135±7	0.69
Potassium (mEq/L)	4.2±0.7	3.9±1.8	0.03
CRP (mg/L)	119.6±5.9	96.±4.3	0.001
Glycemia (mg/dL)	141±62	133±54	0.07
Total bilirubin (mg/dL)	1.5±0.9	0.9±0.4	0.03
Hgb (g/dL)	11.7±2.4	12.5±2.2	<0.001
MCV (fL)	90.9±9.5	91.1±6.4	0.94
RDW (%)	15.6±2.2	14.4±1.9	<0.001
WBC (10^3^/µL)	12.7±8.8	12.4±8.6	0.81
Platelets (10^3^/µL)	190.3±5.5	216±4.3	<0.001
pH	7.39±0.09	7.44±0.07	<0.001
pO_2_ (mmHg)	99.6±32.1	94.2±27.5	0.02
pCO_2_ (mmHg)	32.3±8.8	34.1±9.5	0.02
Arterial HCO_3_ (mEq/L)	20.4±5.1	23.1±4.7	0.001
Arterial lactate (mg/dL)	22.4±3.9	19.1±4.7	0.02
PaO2/FiO_2_	306±116	357±108	<0.001
SAPS 3	57.1±12.7	50.4±11.1	<0.001

AKI: acute kidney injury; CKD: chronic kidney disease; MAP: mean arterial pressure; CRP: C-reactive protein; Hgb: hemoglobin; MCV: mean corpuscular volume; RDW: red blood cell distribution width; WBC: white blood cell; SAPS 3: Simplified Acute Physiology Score 3 prognostic index.

AKI: acute kidney injury.

Higher red blood cell distribution width (%) at intensive care unit admission was found in sepsis-induced AKI Group compared to non-AKI Group (p<0.001).

In sequence with this analysis, table 2 showed that women and higher levels of bicarbonate have a protective effect against the risk of AKI associated with sepsis (around 65% and 12%, respectively). On the other hand, patients with higher levels of urea, RDW, and non-dialysis CKD at admission to the ICU are independently associated with AKI in sepsis.

**Table 2:** Acute kidney injury as response variable and its predictors on admission to intensive care unit

AKI *versus* non-AKI	OR	95%CI for OR		p value
		Lower	Upper	
Urea (mg/dL)	1.047	1.036	1.058	<0.001
Women	0.348	0.224	0.542	<0.001
Bicarbonate (mEq/L)	0.883	0.839	0.930	<0.001
CKD	3.549	1.627	7.743	0.001
RDW (%)	1.158	1.045	1.283	0.005
SAPS 3	1.017	0.996	1.039	0.11
PaO_2_/FiO_2_	0.999	0.997	1.000	0.13
pH	0.149	0.008	2.611	0.19
Arterial lactate (mg/dL)	0.990	0.997	1.000	0.21
Glycemia (mg/dL)	0.998	0.994	1.002	0.33
MAP (mmHg)	0.995	0.984	1.006	0.34
Potassium (mEq/L)	0.933	0.779	1.118	0.45
Age (years)	1.006	0.990	1.022	0.49
Platelets (10^3^/µL)	0.999	0.997	1.002	0.65
CRP (mg/L)	1.000	0.998	1.003	0.79
Hgb (g/dL)	0.999	0.896	1.113	0.98

R^2^= 0.763 (p<0.001); OR: odds ratio; 95%CI: 95% confidence interval; AKI: acute kidney injury; CKD: chronic kidney disease; RDW: red blood cell distribution width; SAPS 3: simplified acute physiology score 3 prognostic index; MAP: mean arterial pressure; CRP: C-reactive protein; Hgb: hemoglobin.

Subsequently, considering the overall outcomes observed during ICU stay of sepsis patients (n=849), it was highlighted: 165 patients (19.4%) required mechanical ventilation in 3.9±2.4 days; 151 patients (17.8%) required red cell transfusion in 8.1±6.4 days; 148 patients (17.4%) required vasopressor drug in 5.1±2.9 days; 73 patients (8.6%) evolved with mortality in 14.2±5.4 days; and 34 patients (4%) required RRT in 9.6±6.9 days after admission to the ICU. An association of RRT requirement with urea [OR 1.023, 95%CI: 1.016-1.030; p<0.001] and RDW [OR 1.180, 95%CI: 1.018-1.368; p=0.03] on ICU admission was also identified. A total of 18 patients (52.9%) in RRT required continuous venovenous hemodiafiltration. The study also found that mortality within 28 days of ICU period had an association with levels of RDW [OR 1.368, 95%CI: 1.221-1.533; p<0.001] and urea [OR 1.011, 95%CI: 1.004-1.057; p=0.001] of admission to ICU.

Finally, a subgroup of patients without CKD (n=759) was analyzed to assess and rule out possible CKD interference. This subgroup of patients without CKD but with sepsis-associated AKI (n=219) had higher levels of RDW (15.4±2.2, 14.4±1.9; p<0.001). There was also an independent association of RDW with sepsis-induced AKI in patients without CKD [OR 1.231, 95%CI: 1.140-1.330; p<0.001].

## DISCUSSION

The main finding of the present study is that RDW is an independent predictor associated with AKI in critically ill patients with sepsis. In addition, the study identified that the highest RDW on admission was associated with the need for RRT during the ICU period.

The patients with sepsis-associated AKI had lower mean arterial pressure and arterial bicarbonate, but with elevated arterial lactate at admission, which symbolizes a situation according to low perfusion and organ dysfunction that often occurs in sepsis.^(17,18)^ Therefore, the relevance of the current study is corroborated by recent studies.^(17,19)^

Sepsis is the most important cause of AKI in the ICU and it is estimated that 15% to 20% of patients with sepsis-associated AKI need RRT.^(17)^ When correlations were analyzed in patients with sepsis, negative associations of bicarbonate with both lactate and creatinine were identified. In addition, a positive correlation of RDW value with variables of poor renal function (urea and creatinine) was found.

The diagnosis of AKI is currently based on increased serum creatinine concentration and/or decreased urine output.^(5,16,19,20)^ Thus, the present study could define and form groups of patients with and without AKI. The group of patients who developed sepsis-associated AKI had lower PaO_2_/FiO_2_ ratio, but higher levels of urea, creatinine, arterial lactate, RDW, and SAPS 3 prognostic index. All these clinical parameters indicate kidney dysfunction, decreased oxygenation, and lower tissue perfusion. Thus, there is more severity and poorer prognosis in patients with AKI associated with sepsis. On the one hand, this study group has recently demonstrated that critically ill patients with COVID-19-related AKI had a lower PaO_2_/FiO_2_ ratio too.^(21)^ Indeed, lung dysfunction from AKI is associated with poorer outcomes.^(22)^ On the other hand, Loveday et al. reported the addition of RDW to APACHE III increased the prognostic value in critically ill patients.^(9)^ Moreover, serum creatinine as a marker of renal function is a variable of the SAPS 3 prognostic index, reflecting greater levels of this prognostic index in patients with AKI.^(9,15)^ Furthermore, the current study found a positive correlation between the prognostic index-SAPS 3 and RDW. This indicates a relationship between RDW and prognostic index for critically ill patients. However, the present results corroborate with the results of other studies.

Moreover, the results of the present study were different from the results of Radovic et al., which observed levels of arterial lactate as an independent predictor of AKI but in patients undergoing cardiac surgery.^(23)^ On the other hand, arterial lactate did not present an independent association with sepsis-induced AKI in the current study. Even so, this study found that the levels of bicarbonate on admission to the ICU were lower in the group that developed AKI. In addition, a multivariate analysis showed that higher levels of arterial bicarbonate have a protective effect on developing AKI. These findings agree with the results of Jung et al., who observed that low levels of serum bicarbonate were associated with a higher incidence of AKI and prolonged ICU stay after cardiac surgery.^(24)^

The RDW is routinely reported as part of a complete blood cell count and expresses variation in the size of circulating erythrocytes.^(7,8,12)^ Red cell distribution width is reported as a marker and predictive value in many clinical settings. Changes and disorders frequently seen in sepsis such as ineffective erythropoiesis or increased destruction of red blood cells cause anemia and consequently greater heterogeneity in the size of these cells and higher RDW.^(7,9,10)^ Moreover, previous studies showed that RDW is associated with AKI in patients with traumatic brain injury, sepsis in pediatric patients, after cardiac surgery, and those with coronary disease.^(25-28)^

The present study confirmed the results found by other researchers. Moreover, as there were higher levels of CRP, arterial lactate, and RDW, in parallel with lower arterial bicarbonate, PaO_2_/FiO_2_ ratio, platelets, and Hgb concentration in patients with sepsis and AKI,^(7,8,17)^ it can be assumed that when inflammation occurs, it reduces iron metabolism, and bone marrow function is inhibited, thus, the erythrocyte proliferation and maturation are inhibited,^(29,30)^ leading to increased RDW values and anemia,^(31)^ while inflammatory dysregulation can reduce tissue perfusion and thus cause AKI.

This study also found that RDW levels were higher in patients who developed AKI and showed that RDW at admission to ICU had an independent association with AKI in multivariate analysis. Interestingly, each 1% of the increase in the RDW value measured was independently associated with important risk (16%) of sepsis-induced AKI. Furthermore, each increase of 1mg/dL in the value of urea (higher than the cut-off of 50mg/dL) at admission was independently associated with a risk of 4% more of sepsis-associated AKI. The current study also found that female sex, and higher serum bicarbonate levels on admission to the ICU were associated with protection against sepsis-induced AKI.

Furthermore, it was also detected that the patients with CKD in stages 1-3, had 3.5 times more likely to develop AKI associated with sepsis independently. Other researchers reported a bilateral interaction of CKD with AKI. Chronic kidney disease predisposes to AKI. On the other hand, severe AKI could worsen the progression of CKD.^(32)^ Even so, the present results showed a relationship between RDW and sepsis-associated AKI also in the subgroup of patients without CKD. Thus, eliminating the interference of CKD in this relationship.

Subsequently, the association of variables at ICU admission independently related to AKI with outcomes within 28 days was also analyzed. Each increase of 1mg/dL in the value of urea higher than the cut-off of 50mg/dL at ICU admission was associated with an increased risk (2.3%) of need for RRT. In turn, each 1% increase in the RDW value measured was associated with the risk (18%) of need for RRT. Also, there was association between mortality within 28 days of ICU stay and both urea and RDW value at ICU admission. Thus, each 1% increase in the measured RDW value was independently associated with 37% likely of mortality at 28 days of ICU stay. Thereby, these results are in robust compliance with other studies. In fact, Oh et al. reported that RDW is a predictor for all-cause mortality in AKI patients on RRT treatment in the ICU.^(33)^ Fernandez et al. reported higher values of RDW as a marker of severity in patients discharged from the ICU.^(34)^

Although intriguing, the present study has some potential limitations. First, the sample evaluated is from a retrospective cohort study. Second, it was in a single-center, there was no intervention by the researchers or sequential analysis of RDW, and there was no pre-specified hypothesis. Finally, given the large number of potential predictors evaluated and the initial lack of selection guided by hypotheses of variables, the possibility of confounding bias cannot be ruled out.

Despite these limitations, one of the main strengths of this study is that it is based on routine blood tests for critically ill patients on admission to the ICU and is inexpensive. Thus, a strong and independent association of RDW with sepsis-induced AKI was found, reflecting severity and organ dysfunction. However, the present study showed that the RDW on ICU admission acts as a predictor of AKI associated with sepsis.

## CONCLUSION

This study concluded that red cell distribution width can be used as a cost-effective marker for sepsis-associated acute kidney injury. Thus, further studies should be conducted to analyze the true predictive value of red blood cell distribution width in sepsis-induced acute kidney injury.

## References

[1] Singbartl K, Kellum JA. AKI in the ICU: definition, epidemiology, risk stratification, and outcomes. Kidney Int. 2012;81(9):819-25. Review. 10.1038/ki.2011.339 21975865

[2] Alobaidi R, Basu RK, Goldstein SL, Bagshaw SM. Sepsis-associated acute kidney injury. Semin Nephrol. 2015;35(1):2-11. Review. 10.1016/j.semnephrol.2015.01.002 PMC4507081 25795495

[3] Poston JT, Koyner JL. Sepsis associated acute kidney injury. BMJ. 2019;364:k4891. Review. 10.1136/bmj.k4891 PMC6890472 30626586

[4] Zarjou A, Agarwal A. Sepsis and acute kidney injury. J Am Soc Nephrol. 2011;22(6):999-1006. Review. 10.1681/ASN.2010050484 21566052

[5] Bellomo R, Kellum JA, Ronco C, Wald R, Martensson J, Maiden M, et al. Acute kidney injury in sepsis. Intensive Care Med. 2017;43(6):816-28. Review. 10.1007/s00134-017-4755-7 28364303

[6] Sun J, Zhang J, Tian J, Virzì GM, Digvijay K, Cueto L, et al. Mitochondria in sepsis-induced AKI. J Am Soc Nephrol. 2019;30(7):1151-61. Review. 10.1681/ASN.2018111126 PMC6622414 31076465

[7] Kim YC, Song JE, Kim EJ, Choi H, Jeong WY, Jung IY, et al. A simple scoring system using the red blood cell distribution width, delta neutrophil index, and platelet count to predict mortality in patients with severe sepsis and septic shock. J Intensive Care Med. 2019;34(2):133-9. 10.1177/0885066618787448 30021478

[8] Wang AY, Ma HP, Kao WF, Tsai SH, Chang CK. Red blood cell distribution width is associated with mortality in elderly patients with sepsis. Am J Emerg Med. 2018;36(6):949-53. 10.1016/j.ajem.2017.10.056 29133071

[9] Loveday S, Sinclair L, Badrick T. Does the addition of RDW improve current ICU scoring systems? Clin Biochem. 2015;48(9):569-74. 10.1016/j.clinbiochem.2015.04.002 25869493

[10] Han YQ, Zhang L, Yan L, Li P, Ouyang PH, Lippi G, et al. Red blood cell distribution width predicts long-term outcomes in sepsis patients admitted to the intensive care unit. Clin Chim Acta. 2018;487:112-6. 10.1016/j.cca.2018.09.019 30218659

[11] Henry BM, Benoit JL, Benoit S, Pulvino C, Berger BA, Olivera MH, et al. Red blood cell distribution width (RDW) predicts COVID-19 severity: a prospective, observational study from the cincinnati SARS-CoV-2 emergency department cohort. Diagnostics (Basel). 2020;10(9):618. 10.3390/diagnostics10090618 PMC7554711 32825629

[12] Jia L, Cui S, Yang J, Jia Q, Hao L, Jia R, et al. Red blood cell distribution width predicts long-term mortality in critically ill patients with acute kidney injury: a retrospective database study. Sci Rep. 2020;10(1):4563. 10.1038/s41598-020-61516-y PMC7067822 32165684

[13] Cho AY, Yoon HJ, Lee KY, Sun IO. Clinical characteristics of sepsis-induced acute kidney injury in patients undergoing continuous renal replacement therapy. Ren. fail. 2018;40(1):403-9. 10.1080/0886022X.2018.1489288 PMC6052425 30015549

[14] Singer M, Deutschman CS, Seymour CW, Shankar-Hari M, Annane D, Bauer M, et al. The Third International Consensus Definitions for Sepsis and Septic Shock (Sepsis-3). JAMA. 2016;315(8):801-10. 10.1001/jama.2016.0287 PMC4968574 26903338

[15] Ko M, Shim M, Lee SM, Kim Y, Yoon S. Performance of APACHE IV in Medical Intensive Care Unit Patients: Comparisons with APACHE II, SAPS 3, and MPM0 III. Acute Crit Care. 2018;33(4):216-21. 10.4266/acc.2018.00178 PMC6849024 31723888

[16] Khwaja A. KDIGO clinical practice Guideline for acute kidney injury. Nephron Clin Pract. 2012;120(4):c179-84. 10.1159/000339789 22890468

[17] Ryoo SM, Lee J, Lee YS, Lee JH, Lim KS, Huh JW, et al. Lactate level versus lactate clearance for predicting mortality in patients with septic shock defined by sepsis-3. Crit Care Med. 2018;46(6):e489-95. 10.1097/CCM.0000000000003030 29432347

[18] Mitra B, Roman C, Charters KE, O’Reilly G, Gantner D, Cameron PA. Lactate, bicarbonate and anion gap for evaluation of patients presenting with sepsis to the emergency department: a prospective cohort study. Emerg Med Australas. 2020;32(1):20-4. 10.1111/1742-6723.13324 31184442

[19] Uchino S, Kellum JA, Bellomo R, Doig GS, Morimatsu H, Morgera S, Schetz M, Tan I, Bouman C, Macedo E, Gibney N, Tolwani A, Ronco C; Beginning and Ending Supportive Therapy for the Kidney (BEST Kidney) Investigators. Acute renal failure in critically ill patients: a multinational, multicenter study. JAMA. 2005;294(7):813-8. 10.1001/jama.294.7.813 16106006

[20] Peerapornratana S, Manrique-Caballero CL, Gómez H, Kellum JA. Acute kidney injury from sepsis: current concepts, epidemiology, pathophysiology, prevention and treatment. Kidney Int. 2019;96(5):1083-99. Review. 10.1016/j.kint.2019.05.026 PMC6920048 31443997

[21] de Almeida DC, Franco M, Dos Santos DR, Santos MC, Maltoni IS, Mascotte F, et al. Acute kidney injury: Incidence, risk factors, and outcomes in severe COVID-19 patients. PloS One. 2021;16(5):e0251048. 10.1371/journal.pone.0251048 PMC8148326 34033655

[22] Lee SA, Cozzi M, Bush EL, Rabb H. Distant Organ Dysfunction in Acute Kidney Injury: a review. Am J Kidney Dis. 2018;72(6):846-56. Review. 10.1053/j.ajkd.2018.03.028 PMC6252108 29866457

[23] Radovic M, Bojic S, Kotur-Stevuljevic J, Lezaic V, Milicic B, Velinovic M, et al. Serum Lactate As Reliable Biomarker of Acute Kidney Injury in low-risk cardiac surgery patients. J Med Biochem. 2019;38(2):118-25. 10.2478/jomb-2018-0018 PMC6411001 30867639

[24] Jung SY, Park JT, Kwon YE, Kim HW, Ryu GW, Lee SA, et al. Preoperative Low Serum Bicarbonate Levels Predict Acute Kidney Injury After Cardiac Surgery. Medicine (Baltimore). 2016;95(13):e3216. 10.1097/MD.0000000000003216 PMC4998548 27043687

[25] Wang RR, He M, Ou XF, Xie XQ, Kang Y. The predictive value of RDW in AKI and mortality in patients with traumatic brain injury. J Clin Lab Anal. 2020;34(9):e23373. 10.1002/jcla.23373 PMC7521248 32844458

[26] Zhang L, Guo KP, Mo Y, Yi SW, Huang CZ, Long CX, et al. [Predictive value of red blood cell distribution width for acute kidney injury in children with sepsis]. Zhongguo Dang Dai Er Ke Za Zhi. 2018;20(7):559-62. Chinese. 10.7499/j.issn.1008-8830.2018.07.009 PMC7389194 30022758

[27] Zou Z, Zhuang Y, Liu L, Shen B, Xu J, Jiang W, et al. Role of elevated red cell distribution width on acute kidney injury patients after cardiac surgery. BMC Cardiovasc Disord. 2018;18(1):166. 10.1186/s12872-018-0903-4 PMC6092813 30107786

[28] Hu Y, Liu H, Fu S, Wan J, Li X. Red blood cell distribution width is an independent predictor of AKI and mortality in patients in the coronary care unit. Kidney Blood Press Res. 2017;42(6):1193-204. 10.1159/000485866 29227977

[29] Semba RD, Patel KV, Ferrucci L, Sun K, Roy CN, Guralnik JM, et al. Serum antioxidants and inflammation predict red cell distribution width in older women: the Women’s Health and Aging Study I. Clin Nutr. 2010;29(5):600-4. 10.1016/j.clnu.2010.03.001 PMC3243048 20334961

[30] Rabb H, Griffin MD, McKay DB, Swaminathan S, Pickkers P, Rosner MH, Kellum JA, Ronco C; Acute Dialysis Quality Initiative Consensus XIII Work Group. Inflammation in AKI: Current Understanding, Key Questions, and Knowledge Gaps. J Am Soc Nephrol. 2016;27(2):371-9. Review. 10.1681/ASN.2015030261 PMC4731128 26561643

[31] Garg AX, Badner N, Bagshaw SM, Cuerden MS, Fergusson DA, Gregory AJ, Hall J, Hare GM, Khanykin B, McGuinness S, Parikh CR, Roshanov PS, Shehata N, Sontrop JM, Syed S, Tagarakis GI, Thorpe KE, Verma S, Wald R, Whitlock RP, Mazer CD; TRICS Investigators and Perioperative Anesthesia Clinical Trials Group. Safety of a Restrictive versus Liberal Approach to Red Blood Cell Transfusion on the Outcome of AKI in Patients Undergoing Cardiac Surgery: a randomized clinical trial. J Am Soc Nephrol. 2019;30(7):1294-304. 10.1681/ASN.2019010004 PMC6622429 31221679

[32] Chawla LS, Eggers PW, Star RA, Kimmel PL. Acute kidney injury and chronic kidney disease as interconnected syndromes. N Engl J Med. 2014;371(1):58-66. Review. 10.1056/NEJMra1214243 PMC9720902 24988558

[33] Oh HJ, Park JT, Kim JK, Yoo DE, Kim SJ, Han SH, et al. Red blood cell distribution width is an independent predictor of mortality in acute kidney injury patients treated with continuous renal replacement therapy. Nephrol Dial Transplant. 2012;27(2):589-94. 10.1093/ndt/gfr307 21712489

[34] Fernandez R, Cano S, Catalan I, Rubio O, Subira C, Masclans J, et al. High red blood cell distribution width as a marker of hospital mortality after ICU discharge: a cohort study. J Intensive Care. 2018;6:74. 10.1186/s40560-018-0343-3 PMC6240256 30473793

